# A Correlation between Inflammatory Factors and Epileptic Seizures: A Meta-analysis

**DOI:** 10.62641/aep.v53i4.1790

**Published:** 2025-08-05

**Authors:** Changqing Cao, Jing Mu, Guiying Hu, Yali Wang, Yigu Gong

**Affiliations:** ^1^Department of Pediatrics, The First Hospital of Lanzhou University, 730000 Lanzhou, Gansu, China

**Keywords:** interleukin-1β, IL-6, TNF-α concentrations, epileptic seizure, relationship, meta-analysis

## Abstract

**Background:**

The pathophysiological mechanisms and relevant biological markers for epileptic seizures largely remained unknown. However, several studies have reported elevated levels of inflammatory factors in the serum of individuals with epileptic seizures. Therefore, this study aims to explore the relationship between inflammatory factors and epileptic seizures.

**Methods:**

We retrieved relevant literature published in various databases, including Embase, PubMed, Cochrane Library, Web of Science, China Wanfang, HowNet, Chinese Biomedical Literature, and VIP, from inception to December 2023. The relationship between inflammatory factors, such as interleukin-1β (IL-1β), interleukin-6 (IL-6), and tumor necrosis factor-alpha (TNF-α), and epileptic seizure was assessed. The selected manuscripts were evaluated based on the predetermined inclusion and exclusion criteria, and relevant data were extracted for meta-analysis using Rev Man 5.0 software (RevMan, Oxford, UK) and Stata 12.

**Results:**

We observed that individuals with epileptic seizures had significantly elevated levels of IL-1β (random-effects model, Standardized Mean Difference (SMD) = 1.87, 95% confidence interval (CI) = [1.17, 2.56], I^2^ = 96.6%, *p* = 0.010), IL-6 (SMD = 1.73, 95% CI = [0.41, 3.05], I^2^ = 96.9%, *p* = 0.010), and TNF-α (random-effects model, SMD = 2.16, 95% CI = [1.13, 3.18], I^2^ = 96.5%, *p* = 0.010). Moreover, the subgroup analysis indicated significant differences between the two groups for the country (origin of publication) (SMD = 1.87, 95% CI = [1.17, 2.56], I^2^ = 96.6%, *p* < 0.001), sampling time (SMD = 1.87, 95% CI = [1.17, 2.56], I^2^ = 96.6%, *p* = 0.010), and sample source (SMD = 1.87, 95% CI = [1.17, 2.56], I^2^ = 96.6%, *p* = 0.010).

**Conclusion:**

The IL-1β, IL-6, and TNF-α levels are increased in individuals with epileptic seizures, which could serve as effective biomarkers for epileptogenesis.

## Introduction

Epilepsy is a neurological disorder characterized by recurrent and unprovoked seizures, affecting individuals of all age groups and ranking among the most common neurological conditions worldwide [1]. The exact etiology of epilepsy often remains unknown, though it can be attributed to various factors such as genetics, brain injury, infections, or developmental disorders [2]. About 50 million people worldwide are living with epilepsy, with a prevalence rate of approximately 0.6–1% of the global population [3]. The incidence of epilepsy tends to be higher in low- and middle-income countries compared to high-income nations [4]. Epilepsy treatment primarily focuses on managing seizures and improving quality of life, typically involving the use of antiepileptic drugs (AEDs) [5]. These medications help reduce the frequency and severity of seizures in most cases. However, surgery may be considered a possible option to remove the specific brain area responsible for the seizures [6]. Diagnosing epilepsy involves a comprehensive evaluation of the person’s medical history, a physical examination, and various diagnostic tests, such as a widely used electroencephalogram (EEG) [7]. However, currently there is a lack of early serological biomarkers for predicting epilepsy. Identifying cost-effective and rapid serological biomarkers is significant in the early diagnosis and prediction of refractory epilepsy, allowing timely intervention and treatment.

Brain inflammation is a crucial mechanism underlying refractory epilepsy. Evidence from extensive clinical and animal-based studies suggests that inflammation may contribute to the development of epilepsy, increase seizure susceptibility, and trigger seizure occurrence [8]. Various inflammatory factors, including interleukin (IL)-1, nuclear factor kappa-B (NF-κB), C-reactive protein, and cyclooxygenase-2 (COX-2), are crucial in the onset of epilepsy by inducing neuronal death and activating astrocytes [9, 10]. Pro-inflammatory cytokines can enhance the release of excitatory neurotransmitters and inhibit the reuptake of glutamate by astrocytes [11, 12]. Lerner and Karelina [13] demonstrated that brain inflammation can induce epilepsy by activating intracellular signaling pathways, leading to abnormal expression or dysfunction of ATP-binding cassette transporter in brain endothelial cells and glial cells of the blood-brain barrier. This dysfunction prevents antiepileptic drugs from reaching the brain parenchyma by pumping them into the capillary lumen, resulting in the development of refractory epilepsy.

Numerous experimental models and clinical studies have associated inflammatory factors, such as interleukin-1β (IL-1β), interleukin-6 (IL-6), and tumor necrosis factor-alpha (TNF-α), with seizure activity; however, some studies are contradictory and have not formed a unified understanding. Moreover, several studies have reported increased expression levels of IL-1β, IL-6, and TNF-α in the plasma and brain tissue of patients during seizures [14, 15, 16]. Conversely, other studies have found no significant changes in these cytokines in the plasma and cerebrospinal fluid of seizure patients [17, 18, 19, 20]. Li *et al*. [21] observed a decrease in IL-1β, IL-6, and TNF-α in the plasma of patients after seizures, whereas patients without seizures showed a slight increase. The exact role of IL-1β, IL-6, and TNF-α in seizure activity remains unclear.

Therefore, we comprehensively explore the relationship between inflammatory factors, such as IL-1β, IL-6, and TNF-α, and epileptic seizure, providing evidence-based insights for early serological predictive biomarkers.

## Methods 

### Inclusion Criteria for the Literature 

The inclusion criteria for relevant literature were as follows: ① The literature indicates the clinical diagnosis of epileptic seizures primarily based on the history of seizures and clinical manifestations, confirmed through evidence such as computed tomographic (CT), magnetic resonance imaging (MRI), and epileptic discharges on electroencephalogram. ② The manuscripts specify the time interval between epileptic seizures and sample collection (≤72 hours), and the method utilized to detect IL-1β, IL-6, and TNF-α. ③ The study includes measurements of cytokine IL-1β, IL-6, and TNF-α in the patient’s plasma, with specific data provided to calculate the concentration of IL-1β. ④ Additionally, healthy individuals’ serum was used as a control, or a baseline control group was included. The MOOSE guidelines checklist is attached **(Supplementary File 1**).

### Exclusion Criteria for the Literature 

Exclusion criteria included: ① Patients with secondary infections or inflammatory diseases within 2 weeks prior to the onset of epilepsy seizures. ② Patients who received immunomodulatory therapy within 6 months prior to the onset of epilepsy seizures. ③ The control group, neurologically and laboratory-based assessment confirmed as normal, must also exclude other neurological disorders. ④ Studies with incomplete clinical data, duplicate publications, review articles, or grey literature where the original article could not be found.

### Data Sources

We identified eligible publications by performing advanced electronic searches across databases, including Wanfang (http://www.wanfangdata.com.cn/), CNKI (https://www.cnki.net/), Chinese Biomedical Literature (https://www.sinomed.ac.cn/), VIP (http://www.cqvip.com/), Embase (https://www.embase.com/), PubMed (https://pubmed.ncbi.nlm.nih.gov/), Cochrane Library (https://www.cochranelibrary.com/), and Web of Science database (https://clarivate.com/webofsciencegroup/solutions/web-of-science/), from their inception to December 2023. The search algorithm included the terms “Interleukin-1β”, “IL-6”, “TNF-α”, and “epileptic seizure” without imposing any language restrictions.

### Data Extraction and Quality Assessment

A preliminary screening was conducted by reviewing the titles and abstracts of the manuscripts. After this, the full texts of the selected articles were thoroughly reviewed for a second screening. Based on the inclusion and exclusion criteria, a final decision was made on whether to include the manuscripts. This process, along with the assessment of research quality, was independently conducted by two researchers. In case of disagreement, the issue was resolved through discussion or with the assistance of a third expert.

The quality of the selected manuscript was evaluated and scored using the “Quality Evaluation and Scoring Table for Non-Randomized Controlled Clinical Trials”. Scores were assigned based on criteria such as diagnostic standards, baseline variables control, and confounding factors management [22].

### Statistical Analysis

Statistical analysis was performed using Rev Man 5.0 software (RevMan, Oxford, UK) and Stata12 (STATA Corporation, College Station, Texas, USA), provided by the Cochrane Collaboration. Heterogeneity was assessed using the χ^2^ test. If no statistical heterogeneity was observed among the studies (*p* > 0.05, I^2^ < 50%), a fixed-effects model was used for meta-analysis. If statistical heterogeneity was found (*p* < 0.05, I^2^ > 50%), a random-effects model was applied. The standardized mean difference (SMD) was used as the effect size, with a 95% confidence interval (CI) calculated at a significance level of α = 0.05.

## Results

### Characteristics of Selected Literature

**Fig. 1.**
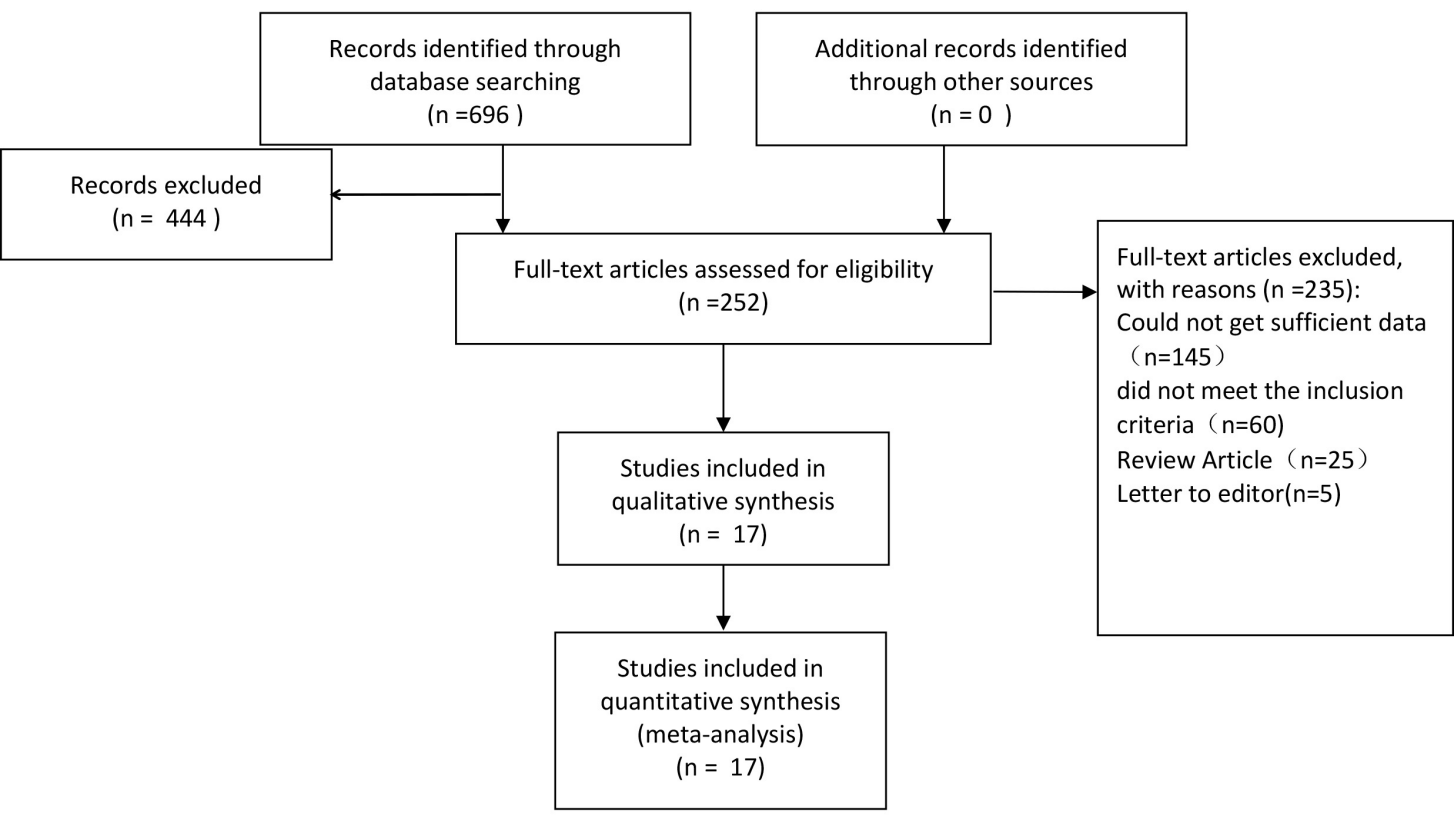
**A flow diagram of the literature search process**.

**Fig. 2.**
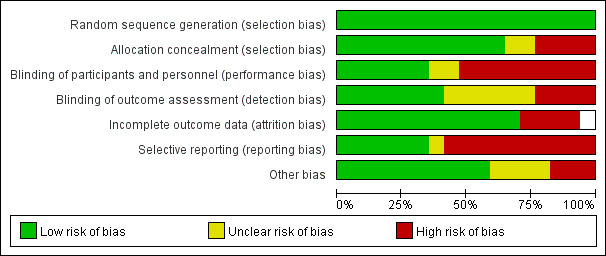
**The risk of bias: review authors’ judgements about each risk of bias item for included studies**.

Out of 696 studies initially identified, 444 potentially relevant studies were extracted after excluding duplicates. Furthermore, we excluded review articles, case reports, letters, and studies that didn’t meet the inclusion criteria, leaving 17 studies for meta-analysis [23, 24, 25, 26, 27, 28, 29, 30, 31, 32, 33, 34, 35, 36, 37, 38, 39]. The study selection process is depicted in Fig. 1. The characteristics of these 17 studies, published between 2000 and 2024, are shown in Table 1 (Ref. [23, 24, 25, 26, 27, 28, 29, 30, 31, 32, 33, 34, 35, 36, 37, 38, 39]). These studies were conducted in China, Finland, Germany, and Egypt. Additionally, the overall quality of these studies was moderate (Fig. 2).

**Table 1.** **Basic characteristics of the included literature**.

First author/year	Country	Subjects (EG/CG)	Sampling time (h)	Research type	NOS score	Sample source	Disease course (year)
El-Kammah 2022 [23]	Egypt	30/50	<24	Case-control research	6	Plasma	5∼16
Fu 2017 [24]	China	59/30	<48	Case-control research	7	Plasma	Unclear
Gu 2022 [25]	China	44/59	<48	Case-control research	8	Serum	2.5∼8
Hu 2018 [26]	China	32/30	<24	Case-control research	6	Serum	1∼20
Hulkkonen 2004 [27]	Finland	10/400	<48	Case-control research	8	Plasma	17∼63
Zhao 2024 [28]	China	40/40	<48	Case-control research	6	Serum	Unclear
Jing 2014 [29]	China	53/30	<48	Case-control research	7	Serum	0.5∼23
Peltola 2000 [30]	Finland	22/18	<24	Case-control research	8	Plasma	Unclear
Lehtimäki 2007 [31]	Finland	12/8	<24	Case-control research	7	Plasma	8∼38
Lai 2021 [32]	China	100/30	<48	Case-control research	7	Serum	≤3
Li 2018 [33]	China	100/100	<48	Case-control research	6	Serum	0.3∼20
Li 2023 [34]	China	108/40	<24	Case-control research	6	Serum	Unclear
Qiao 2014 [35]	China	60/50	<48	Case-control research	6	Plasma	Unclear
Bauer 2009 [36]	Germany	18/25	<24	Case-control research	7	Plasma	21 ± 14
Alapirtti 2009 [37]	Finland	20/20	<24	Case-control research	8	Plasma	2∼52
Kazemian 2022 [38]	Iran	24/20	<48	Case-control research	7	Serum	0.1∼9
Zheng 2017 [39]	China	61/50	<48	Case-control research	7	Serum	1.6∼22

Note: EG, the experimental group; CG, the control group; NOS, Newcastle-Ottawa Scale.

### Forest Plot of IL-1β Concentrations between Epilepsy Patients with Seizures and Normal Controls

**Fig. 3.**
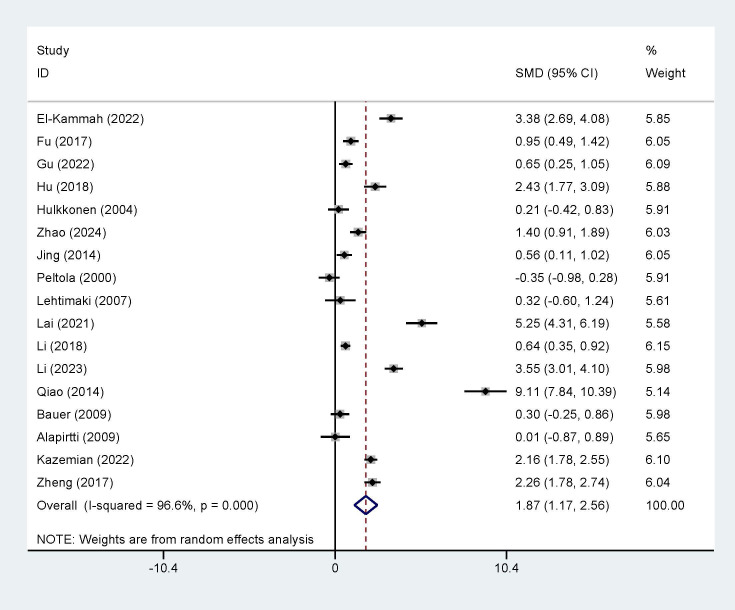
**Forest plot of interleukin (IL)-1β concentrations between patients with epilepsy seizures and normal controls**. SMD, Standardized Mean Difference; CI, confidence interval.

The meta-analysis of data from all eligible studies showed that IL-1β concentrations were significantly high in individuals with epileptic seizures (random-effects model, Standardized Mean Difference (SMD) = 1.87, 95% CI = [1.17, 2.56], I^2^ = 96.6%, *p* < 0.0001, Fig. 3), indicating a statistically significant difference between the two groups.

### Forest Plot of TNF-α Concentrations between Patients with Epilepsy Seizures and Normal Controls

**Fig. 4.**
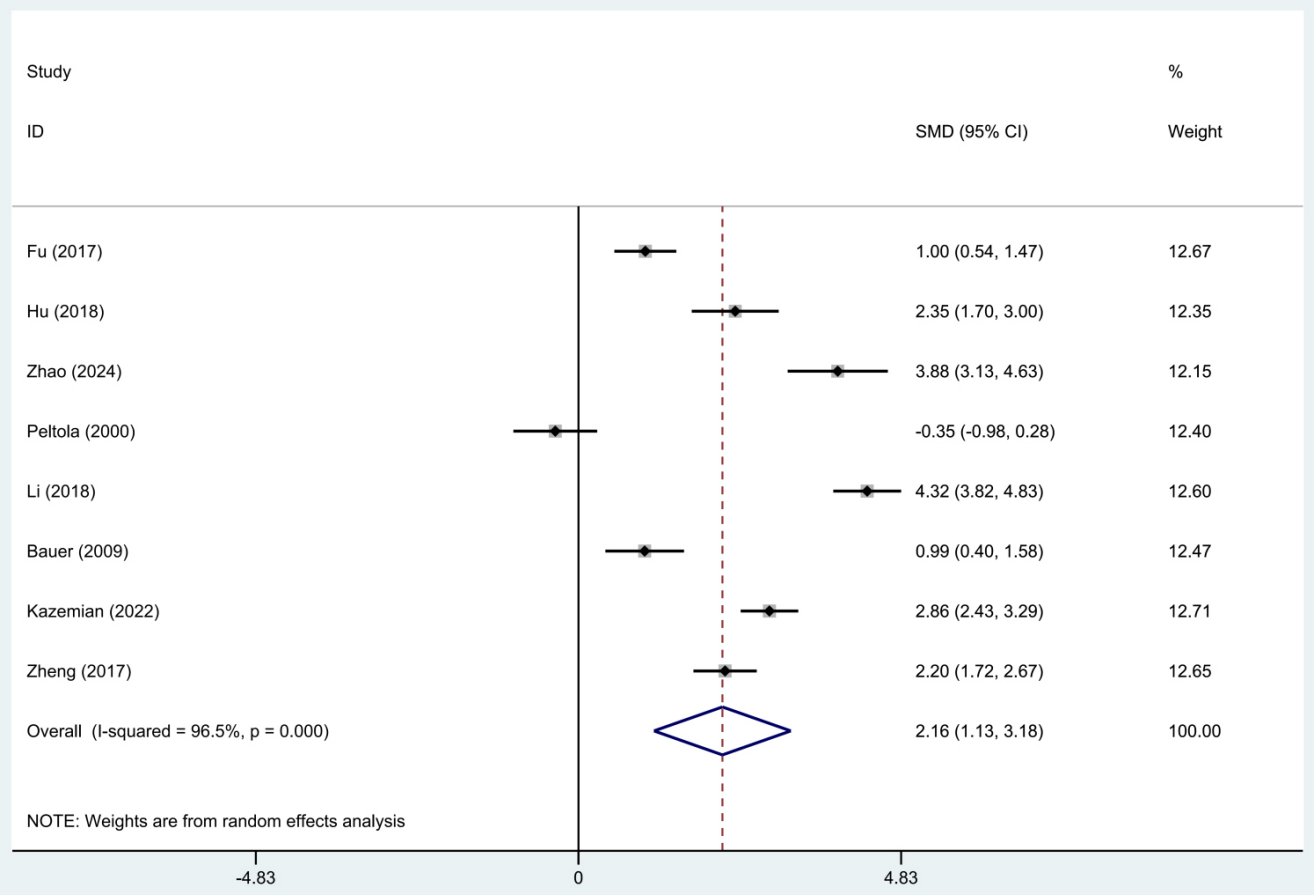
**Forest plot of TNF-α concentrations between individuals with epilepsy seizures and normal controls**.

The meta-analysis of data from eight eligible studies revealed that TNF-α concentrations were significantly elevated in individuals with epileptic seizures (random-effects model, SMD = 2.16, 95% CI = [1.13, 3.18], I^2^ = 96.5%, *p* < 0.0001, Fig. 4), indicating a statistically significant difference between the two groups.

### Forest Plot of IL-6 Concentrations between Patients with Epilepsy Seizures and Normal Controls

**Fig. 5.**
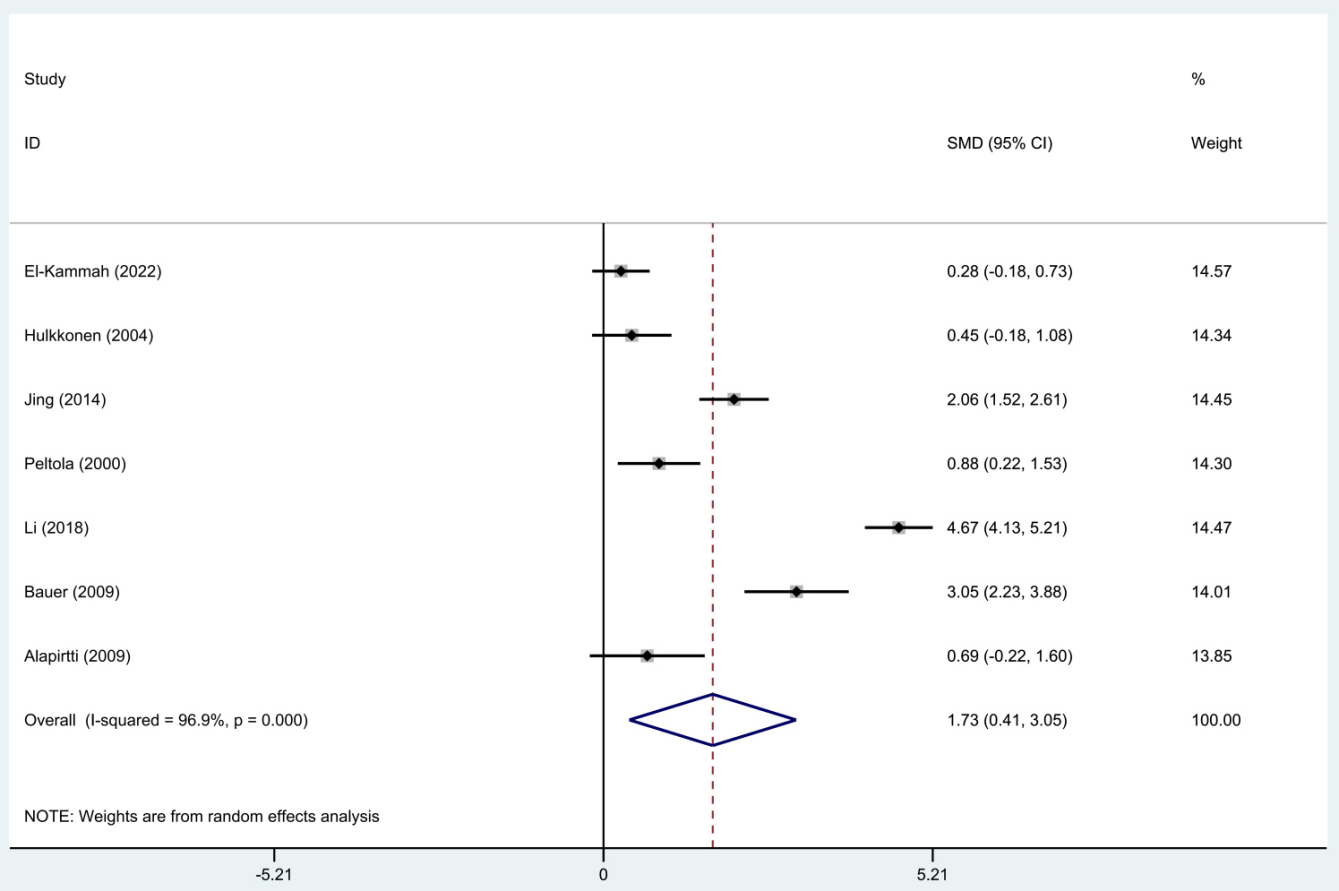
**Forest plot of IL-6 concentrations between individuals with epilepsy seizures and normal controls**.

Compared to the control group, individuals with epileptic seizures had significantly higher IL-6 concentrations (SMD = 1.73, 95% CI = [0.41, 3.05], I^2^ = 96.9%, *p* =0.010, Fig. 5), which indicating a statistically significant difference between the two groups.

### Subgroup Analysis and Publication Bias

**Fig. 6.**
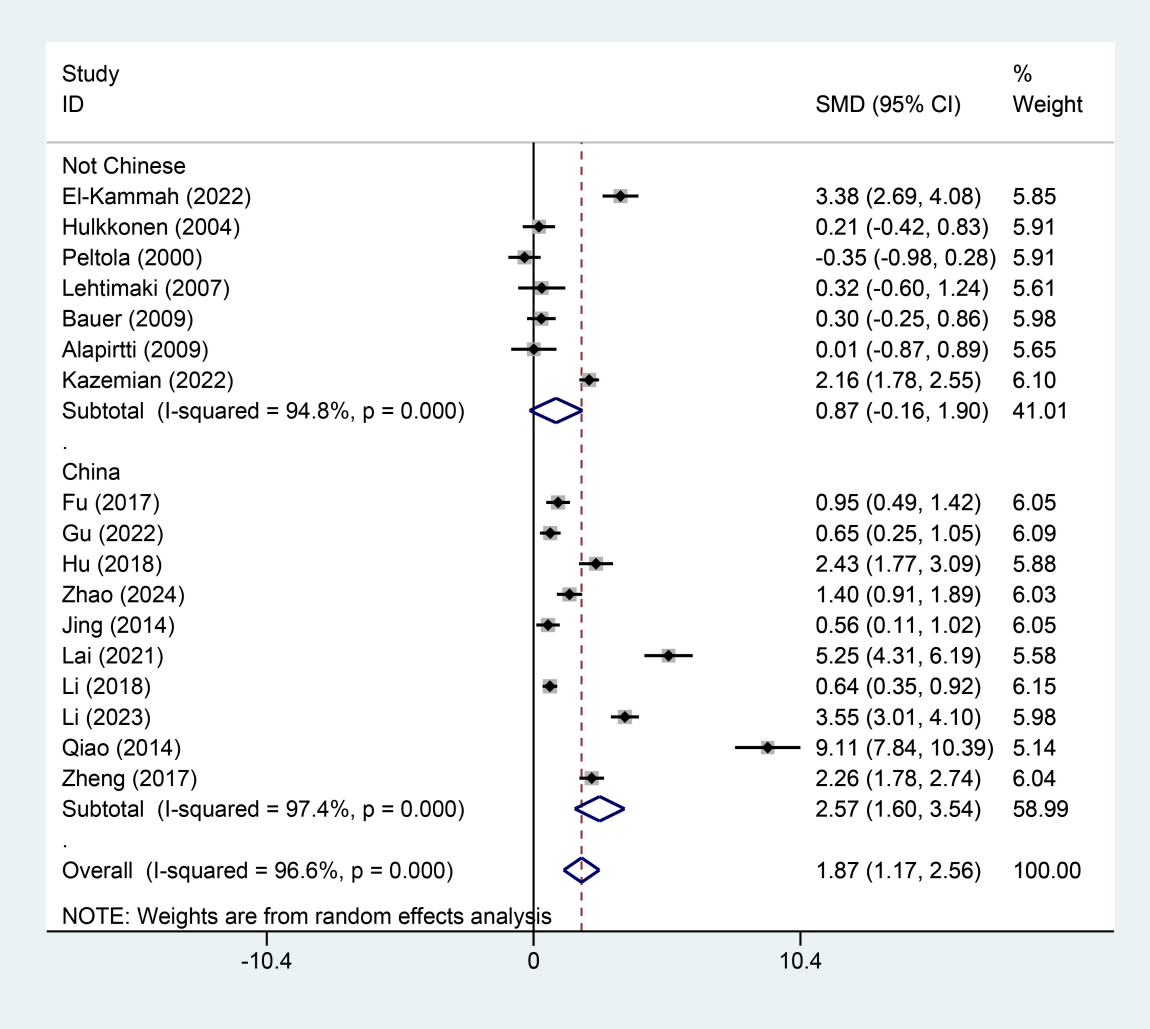
**Subgroup analysis based on origin of publication (country)**.

**Fig. 7.**
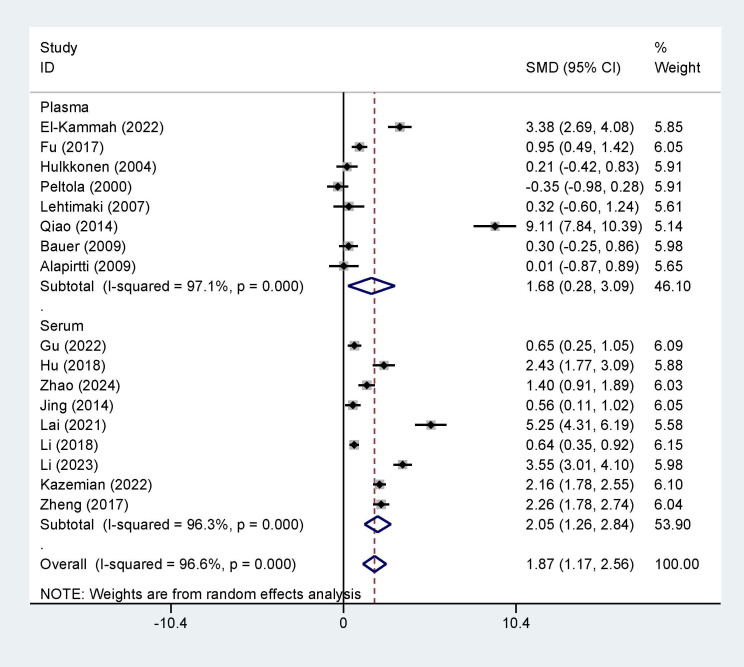
**Subgroup analysis based on sample source**.

**Fig. 8.**
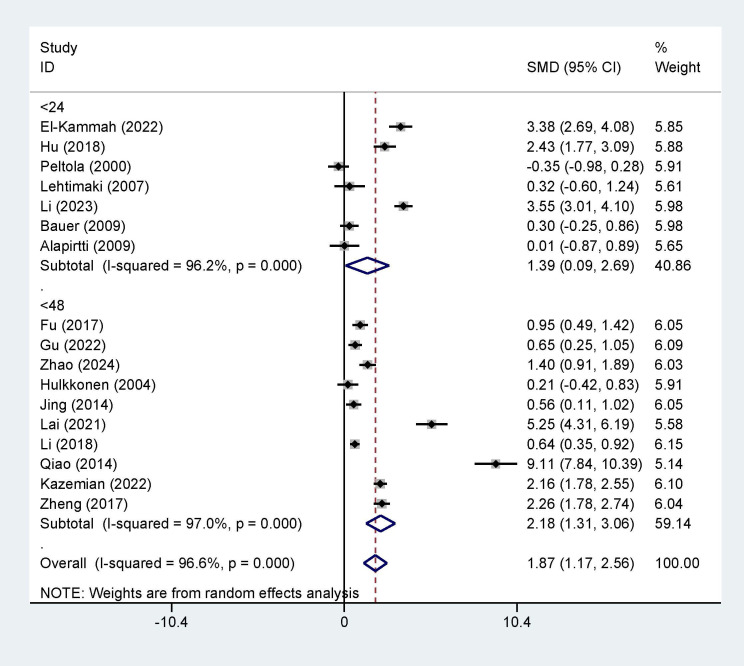
**Subgroup analysis based on sampling time (h)**.

**Fig. 9.**
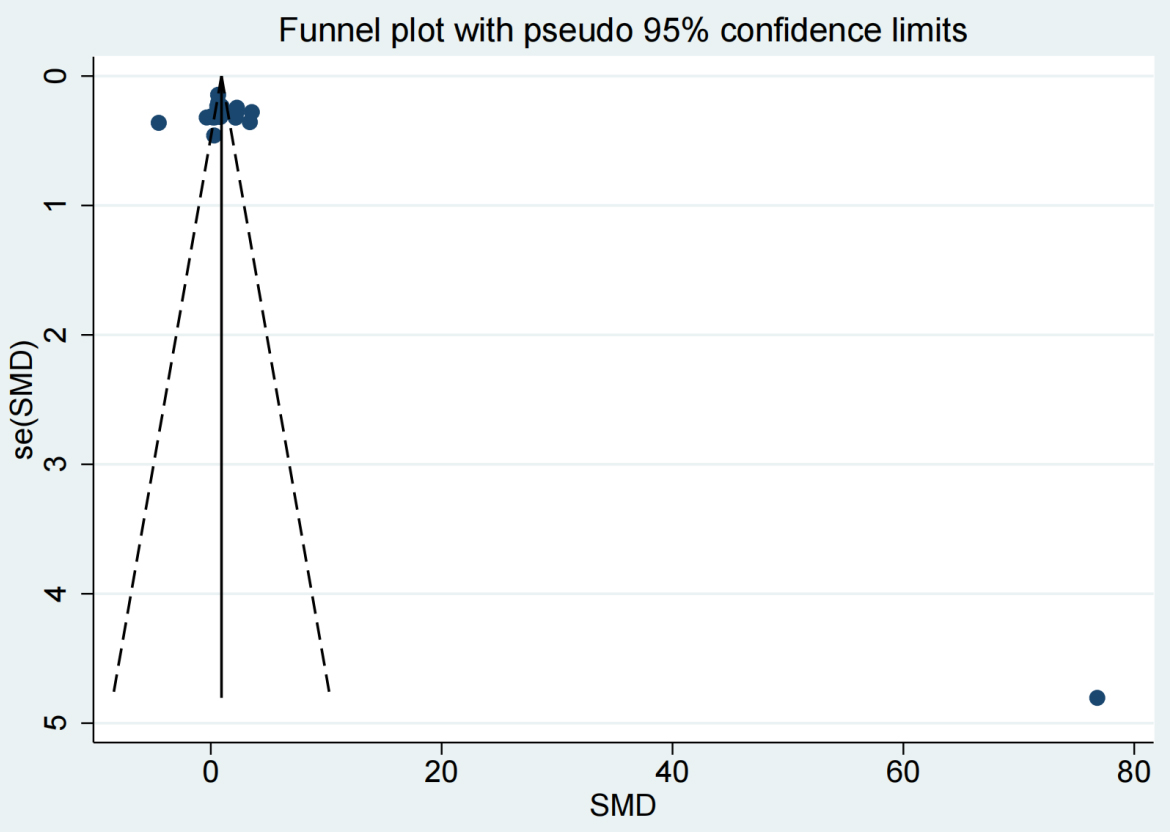
**Funnel plot analysis of publication bias**.

Furthermore, we conducted a subgroup analysis comparing variables such as country (origin of publication), sampling time, and sample source. The results showed significant differences between the two groups for the country (SMD = 1.87, 95% CI = [1.17, 2.56], I^2^ = 96.6%, *p* < 0.001, Fig. 6), sample source (SMD = 1.87, 95% CI = [1.17, 2.56], I^2^ = 96.6%, *p* < 0.0001, Fig. 7), and sample time (SMD = 1.87, 95% CI = [1.17, 2.56], I^2^ = 96.6%, *p* < 0.0001, Fig. 8). Additionally, the funnel plots (Fig. 9) for IL-1β concentrations demonstrated no publication bias.

## Discussion

We found a significant correlation between serum IL-1β levels and seizure occurrence [40]. This analysis is based on the reason that increased expression of IL-1β can stimulate glial cells to produce various cytokines, such as tumor necrosis factor-α and interleukins, which have neurotoxic effects [41]. Furthermore, IL-1β is predominantly distributed in the hippocampus of the brain [42], where its inflammatory and immune responses play a crucial role in epilepsy. Furthermore, stimulation from antiepileptic drugs and excitatory toxins have been found to induce IL-1β production in hippocampal astrocytes. Both endogenous and exogenous IL-1β can enhance seizure activity by prolonging abnormal discharges [43].

Cytokines are crucial for neuronal survival. Research has found that IL-1β can play a protective and nourishing role in neurons [44]. However, a research reported that increased levels of IL-1β can exacerbate brain damage in individuals with local ischemia, trauma, or excitotoxicity [45]. Moreover, experimental studies have indicated that seizure-induced cytokine-mediated signaling pathways may lead to neuronal apoptosis or cell death [46]. The pro-epileptic and antiepileptic effects of cytokines often depend on their concentration, duration, and complex interactions with other inflammatory factors [47]. Therefore, the role of IL-1β should be determined in conjunction with changes in other inflammatory factors. Further extensive research is needed to elucidate the specific mechanisms of IL-1β in the pathogenesis of epilepsy.

IL-6 and TNF-α are two pro-inflammatory cytokines implicated in the pathogenesis of epilepsy. Studies have demonstrated elevated levels of IL-6 and TNF-α in the brains of individuals with epilepsy, suggesting that these cytokines may play a role in the development and progression of the disorder [48, 49, 50]. IL-6 and TNF-α are known to promote neuroinflammation and improve neuronal excitability, which are critical factors in seizure development [51]. Moreover, these cytokines have been shown to disrupt the blood-brain barrier, leading to increased permeability and allowing for the infiltration of inflammatory cells into the brain [52]. Furthermore, IL-6 and TNF-α have been shown to modulate the activity of neurotransmitters and ion channels in the brain, which can further contribute to seizure development [53]. Overall, the relationship between IL-6, TNF-α, and epilepsy is complex and multifaceted, demanding further research to fully elucidate the mechanisms by which these cytokines contribute to the disorder.

The heterogeneity observed in the results of inflammatory factors between studies is substantial and warrants detailed discussion. This variability can be attributed to several factors, including differences in patient demographics (e.g., age, sex, epilepsy subtype), disease severity, and the methodology used in the included studies (e.g., assay techniques, sample sizes). Additionally, other confounding variables such as the use of antiepileptic drugs, comorbidities, and environmental factors could influence the inflammatory markers observed. Despite these variations, the consistent elevation of IL-1β, IL-6, and TNF-α levels across most studies strongly suggests a significant role of neuroinflammation in epilepsy. Further research is needed to standardize experimental protocols and account for these sources of heterogeneity to improve the reliability and generalizability of the findings.

## Conclusion

In conclusion, the levels of IL-1β, IL-6, and TNF-α are increased in individuals with epileptic seizures. Our meta-analysis suggests that this systemic inflammatory response, characterized by elevated levels of IL-6, IL-1β, and TNF-α, may serve as effective biomarkers for epileptogenesis and could contribute to the onset of disease.

## Availability of Data and Materials

The inquiries of original contributions presented in the study can be directed to the corresponding author.

## Supplementary Material

Supplementary material associated with this article can be found, in the online version, at https://doi.org/10.62641/aep.v53i4.1790.
